# Transdiagnostic Efficacy of Cariprazine: A Systematic Review and Meta-Analysis of Efficacy Across Ten Symptom Domains

**DOI:** 10.3390/ph18070995

**Published:** 2025-07-02

**Authors:** Agota Barabassy, Réka Csehi, Zsófia Borbála Dombi, Balázs Szatmári, Thomas Brevig, György Németh

**Affiliations:** Global Medical Division, Gedeon Richter Plc, 1103 Budapest, Hungary

**Keywords:** transdiagnostic, partial agonist, cariprazine

## Abstract

**Introduction**: The introduction of the transdiagnostic approach in psychiatry shifts the focus from discrete diagnoses to shared symptoms across various disorders. The Transdiagnostic Global Impression—Psychopathology (TGI-P) scale is a newly developed tool designed to assess psychiatric symptoms across diagnostic boundaries. It evaluates ten core symptom domains—positive, negative, cognitive, manic, depressive, addiction, anxiety, sleep, hostility, and self-harm—regardless of specific diagnoses. Objective: This study aims to evaluate the efficacy of cariprazine across these ten transdiagnostic symptom domains. **Methods**: A systematic literature review and meta-analysis were conducted in accordance with the Preferred Reporting Items for Systematic Reviews and Meta-Analyses (PRISMA) guidelines. Searches were performed on EMBASE and clinicaltrials.gov. Efficacy measures such as the Positive and Negative Syndrome Scale (PANSS), Montgomery–Åsberg Depression Rating Scale (MADRS), Young Mania Rating Scale (YMRS), Hamilton Anxiety Rating Scale (HAM-A), and Columbia-Suicide Severity Rating Scale (C-SSRS) were used to assess cariprazine’s effect on the ten transdiagnostic symptoms. Multilevel random-effects meta-analyses were conducted to evaluate the efficacy of cariprazine versus placebo in alleviating depressive and anxiety symptoms across clinical trials. **Results**: A total of 30 studies were included in the review. Cariprazine showed therapeutic benefits on positive, negative, manic, and depressive symptoms in specifically designed trials. Preliminary positive effects were seen on anxiety, hostility, and cognitive symptoms across disorders. However, specific trials have not been conducted for anxiety disorders or cognitive impairment. Meta-analyses demonstrated that cariprazine significantly reduces both depressive and anxiety symptoms compared to placebo. Cariprazine significantly improved sleep-related symptoms in both mania and depression trials. Suicidality was evaluated in non-suicidal populations, and no increase was observed. Addiction symptoms were part of the exclusion criteria in the RCTs, so they could not be assessed. Previous reports of cariprazine’s anti-craving and anti-abuse effects come from real-world evidence rather than RCT data. **Conclusions**: Cariprazine appears to be promising in addressing a broad range of symptom domains across psychiatric conditions.

## 1. Introduction

Psychiatric diagnosis relies heavily on classification systems like the Diagnostic and Statistical Manual of Mental Disorders (DSM, 5th edition) [1] and the International Classification of Diseases (ICD, 11th edition) [2], which organize symptoms into discrete diagnostic categories. While these systems have a global impact on the understanding, assessment, and treatment of mental disorders [3,4], they often oversimplify mental health conditions by enforcing rigid diagnostic boundaries. Individuals frequently present with overlapping symptoms that span multiple diagnoses, resulting in high rates of comorbidity and diagnostic inconsistency [3]. Additionally, people with the same diagnosis can present with entirely different symptom profiles and severity levels [3].

These limitations have prompted growing interest in transdiagnostic approaches, which focus on shared mechanisms and symptom dimensions across psychiatric disorders [3]. Such approaches aim to move beyond fixed diagnostic labels, supporting more individualized, flexible care and a deeper understanding of the core drivers of mental distress.

Despite increasing research activity in this area [4], transdiagnostic frameworks are yet to be translated into validated tools with practical clinical utility [5]. Ambiguity in the definition of “transdiagnostic” further contributes to inconsistent application in both research and practice—most commonly, “transdiagnostic” is used to stress the aspect of “across physical and mental health diagnoses” or “overarching symptoms” [4,5].

Recent transdiagnostic research, including a 2024 large-scale AI-driven study, has identified recurring symptom clusters such as depression, anxiety, psychosis, addiction, and self-harm that cut across diagnostic boundaries [3,6]. Other studies point to common transdiagnostic symptom domains such as mood, anxiety, agitation, and sleep disturbances [1,2,7]. Despite the availability of well-established, validated scales for specific diagnoses [e.g., Positive and Negative Syndrome Scale (PANSS) for schizophrenia, Montgomery-Åsberg Depression Rating Scale (MADRS) for depression, Young Mania Rating Scale (YMRS) for bipolar mania, Hamilton Anxiety Rating Scale (HAM-A) for anxiety symptoms, and the Hamilton Depression Rating Scale (HAM-D) for depressive disorders], there is still no widely accepted instrument for measuring transdiagnostic processes. Existing frameworks, like the Research Domain Criteria (RDoC), the Hierarchical Taxonomy of Psychopathology (HiTOP), and clinical staging models, offer useful concepts but are not designed for practical clinical use, highlighting the need for clear, scalable tools to track symptoms across disorders in real-world settings [8]. A new scale, known as the Transdiagnostic Global Impression—Psychopathology scale (TGI-P), has been recently developed [9] to provide a user-friendly tool for measuring patients’ symptoms across various disorders. Ten transdiagnostic symptoms were included in the scale (positive, negative, manic, depressive, addiction, cognitive, anxiety, sleep, hostility, as well as self-harm symptoms) that were defined using deductive approaches. A comprehensive overview of the symptom domains assessed by the scale can be found in the original publication by Correll and colleagues [9].

It is important to note, however, that transdiagnostic assessment is only one side of the coin—equally or even more important is the treatment that follows. In everyday clinical practice, treatment decisions often culminate in the prescription of medications [6]; and while on one hand, there are existing treatments that target specific, well-defined symptoms across disorders such as anxiety or insomnia, it would be ideal to have drugs that have the ability to address multiple symptoms independent from diagnosis. One treatment that has been approved across multiple disorders is cariprazine, which was approved globally for schizophrenia and bipolar I disorder—including manic/mixed and depressive episodes—and as an adjunctive treatment for major depressive disorder [10]. The therapeutic effect of cariprazine across diverse symptom domains may be attributed to its specific receptor profile: it acts as a partial agonist at dopamine D_3_, D_2_, and serotonin 5-HT_1A_ receptors, and as an antagonist at serotonin 5-HT_2A_, 5-HT_2B_, and histamine H_1_ receptors [11,12]. Among antipsychotics, cariprazine has the highest affinity to the D_3_ receptors. The lower affinities of other antipsychotics for the D_3_ receptor relative to the very high affinity of dopamine itself mean that in the living brain, the D_3_ receptor is not blocked by any antipsychotic other than cariprazine [12,13]. Furthermore, cariprazine has only a low affinity for serotonin 5-HT_2C_ and adrenergic α1 receptors [12]. Based on the receptor-binding profile and the activity of cariprazine at these receptors, one would expect efficacy on positive [14] and mania [14,15] symptoms (D_2_ effect), along with effects on negative, cognitive and addiction symptoms (D_3_ effect [16,17,18,19,20,21,22], 5-HT_1A_ [23]), improvement of mood (D_3_, 5-HT_2C_ [24,25]) and anxiolytic effects based on 5-HT_1A_ [24].

Given its broad receptor profile and effects across multiple symptom domains, cariprazine may offer transdiagnostic therapeutic benefits. Therefore, the aim of the present study is to examine the transdiagnostic effects of cariprazine on the ten symptom domains, previously identified by the TGI-P scale, namely positive, negative, cognitive, manic, depressive, addiction, anxiety, sleep, hostility, and self-harm symptoms across various disorders.

## 2. Methods

### 2.1. Search Criteria

To examine the clinical efficacy of cariprazine across symptoms, a systematic literature review focusing on randomized clinical trials (RCTs) was performed in accordance with the preferred reporting items for systematic reviews and meta-analyses (PRISMA) statement [26]. Searches were performed on EMBASE using the keywords “cariprazine”, “major topic”, “randomized controlled trial”, and “non-conference material”, screening for cariprazine in the title or abstract. Additionally, the clinicaltrials.gov register was searched with the terms “cariprazine”, “Phase: 2, 3, 4”, “Interventional”, and “Studies with results”. The searches were limited to studies published until December 2024. Full-text articles were reviewed for eligibility based on predefined inclusion and exclusion criteria.

### 2.2. Inclusion and Exclusion Criteria

The inclusion criteria were as follows: (1) only RCTs specifically reporting on the efficacy cariprazine in adult populations with psychiatric disorders were considered; (2) post hoc analyses of these RCTs reporting new efficacy data were included if they addressed the research questions; and (3) only English-language works were considered. The exclusion criteria were the following: (1) records focusing on other aspects of cariprazine treatment (e.g., safety, dosing, switching, pharmacokinetics, drug–drug interaction, formulations, health economics); (2) records reporting the same efficacy data in different subpopulations (e.g., by race, age, sex, adolescents, elderly); and (3) studies not providing sufficient data or not addressing the research questions (i.e., efficacy of cariprazine in treating transdiagnostic symptom clusters).

### 2.3. Recorded Variables and Data Extraction

Data extraction was performed independently by two of the authors (A.B. and R.C.). The following variables were recorded from each included study for the qualitative analysis: author(s), year of publication, study code, study design, title of study, clinical condition (diagnosis according to DSM), and transdiagnostic symptom(s) addressed. Additionally, population characteristics, intervention details, and outcome measures were recorded. For the quantitative part, the meta-analysis, least square (LS) mean changes from baseline to endpoint for the cariprazine and placebo arms, along with the corresponding standard errors (SEs), LS mean differences between cariprazine and placebo, and their respective SEs and 95% confidence intervals (CIs), were tabulated.

### 2.4. Outcome Measures

For each study, outcome parameters were systematically identified, including all reported primary, secondary, and additional endpoints, and were mapped to transdiagnostic symptom domains according to Table 1 (for the methodology, see Supplementary Material—Table S1). Where possible and available, data from pooled studies was favored over data from single studies as these enhance statistical power, improve precision, and allow for the detection of nuances that individual studies might miss. Any disagreements between reviewers regarding the selection or prioritization of outcome measures, when multiple options were available, were resolved through discussion or, if needed, by consulting a third reviewer (Z.B.D.). When necessary, co-authors (T.B., G.N., B.S.) were contacted to clarify missing or unclear data.

#### 2.4.1. Positive and Negative Syndrome Scale

The PANSS is a clinician-rated tool used to measure the severity of symptoms of schizophrenia [27]. The scale consists of 30 items, each rated on a scale from 1 to 7. The PANSS Factor Scores by Marder (Table 2) are widely accepted to better assess and target specific symptom domains of schizophrenia than the sub-scores [28]. Therefore, wherever available, PANSS Factor Scores were used to assess the transdiagnostic symptoms instead of the PANSS Total or sub-scores.

#### 2.4.2. Montgomery–Åsberg Depression Rating Scale

The MADRS is a clinical assessment tool used to measure the severity of depressive episodes in adults [29]. It consists of 10 items, each rated on a scale from 0 to 6, with higher scores indicating more severe depression. The items cover various symptoms of depression, such as sadness, tension, sleep disturbances, and appetite changes. The MADRS is widely used in both clinical practice and research to evaluate treatment outcomes and monitor changes in depressive symptoms over time.

#### 2.4.3. Young Mania Rating Scale

The YMRS is a clinical assessment tool designed to evaluate the severity of manic symptoms [30]. Developed by Robert Young and colleagues, the YMRS consists of 11 items that assess various aspects of mania, such as elevated mood, increased motor activity, sexual interest, sleep patterns, irritability, and speech. Each item is rated on a scale, with some items ranging from 0 to 4 and others from 0 to 8, allowing for a nuanced measurement of symptoms. The total score can range from 0 to 60, with higher scores indicating more severe manic symptoms.

#### 2.4.4. Negative Symptom Assessment-16

The Negative Symptom Assessment-16 (NSA-16) is a clinician-rated instrument measuring the severity of negative symptoms in schizophrenia across five domains: blunted affect, alogia, anhedonia, avolition, and social withdrawal. It includes 16 items rated on a 6-point scale, with higher scores indicating greater symptom severity.

#### 2.4.5. Hamilton Anxiety Rating Scale

The HAM-A is one of the first rating scales developed to measure the severity of anxiety symptoms [31]. Created by Max Hamilton in 1959, the HAM-A consists of 14 items that assess both psychic anxiety (mental agitation and psychological distress) and somatic anxiety (physical complaints related to anxiety). Each item is rated on a scale from 0 (not present) to 4 (severe), with total scores ranging from 0 to 56.

#### 2.4.6. Columbia-Suicide Severity Rating Scale

The Columbia-Suicide Severity Rating Scale (C-SSRS) is a tool used to assess the severity and immediacy of suicide risk. Developed by researchers at Columbia University, the University of Pennsylvania, and the University of Pittsburgh, the C-SSRS evaluates both suicidal ideation and behavior through a series of structured questions [32]. These questions cover aspects such as the presence and intensity of suicidal thoughts, the planning and preparation for suicide attempts, and the history of suicidal behavior.

#### 2.4.7. Functioning Assessment Short Test

The Functioning Assessment Short Test (FAST) is a widely used tool in psychiatry, particularly for assessing functional impairment in patients with bipolar disorder [33]. This 24-item scale evaluates six areas of functioning: autonomy, occupational functioning, cognitive functioning, financial issues, interpersonal relationships, and leisure time.

#### 2.4.8. Cognitive Drug Research Battery

The Cognitive Drug Research Battery (CDR) System is a computerized battery of cognitive tests designed to assess various aspects of cognitive function, including attention [34]. Developed in the late 1970s, the CDR System is widely used in clinical trials to measure the effects of drugs on cognitive performance.

#### 2.4.9. Color Trail Test

Color Trail Test (CTT) is a culture-fair, performance-based neuropsychological test assessing sustained attention, sequencing, mental flexibility, and visual-motor tracking. It consists of two parts (CTT-1 and CTT-2), where participants connect numbered colored circles in sequence under increasing cognitive demands.

### 2.5. Data Analyses

Meta-analyses were conducted to explore parameters for which the direction or consistency of evidence appeared ambiguous or inconclusive based on the initial qualitative synthesis. These were depressive and anxiety symptoms. Eligible studies included those reporting LS mean differences from baseline to end of the observational period comparing cariprazine to placebo. For depression, the MADRS Total Score provided information on core depressive symptomatology. For anxiety, the HAM-A Total Scores were included. Many studies included multiple cariprazine dose arms compared to a shared placebo group. To account for the statistical dependency arising from these shared comparators, a multilevel (hierarchical) random-effects meta-analytic model was used, with study ID modelled as a random factor. Analyses were conducted in RStudio (version 2024.04.2) using the metafor package. Results were reported as pooled LS mean differences with corresponding standard errors and 95% confidence intervals and visualized using forest plots.

To interpret the efficacy of cariprazine on transdiagnostic symptoms, three categories for the strength of evidence were defined: Strong evidence was defined as the presence of randomized controlled trials (RCTs) specifically designed to evaluate the symptom domain in question, conducted in a relevant and vulnerable patient population. Moderate evidence was assumed if findings were consistent across disorders but were derived from trials not specifically targeting the symptom domain or symptom-specific populations, or if symptom measurement relied on non-specific scales. Weak evidence was assumed when data came from non-RCTs or when limitations in design or measurement reduced interpretability.

## 3. Results

### 3.1. PRISMA Flowchart

**Figure 1:**
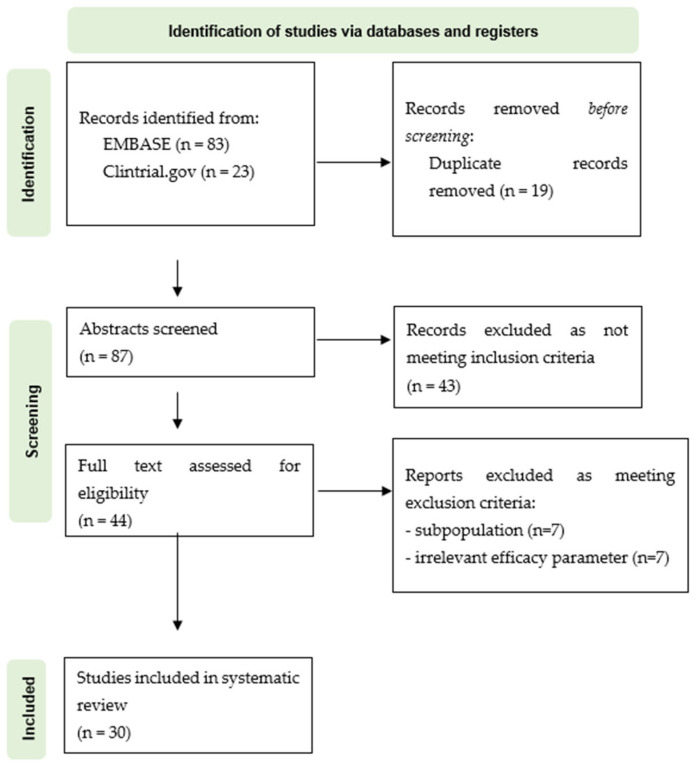
PRISMA flowchart of the systematic review.

The search identified 106 articles that were screened for eligibility after removing duplicates. Among the articles retrieved, 30 met the eligibility criteria. The PRISMA flowchart is shown in Figure 1, and the studies are summarized in Table 3.

### 3.2. Study Characteristics

Studies were performed in the indications of schizophrenia (including a study in patients with persistent, predominant, and primary negative symptoms of schizophrenia), bipolar I mania, bipolar I depression, and major depression as add-on treatment to antidepressants. These were multicenter, multinational, randomized, double-blind, placebo- or active-controlled, parallel-group studies.

Cariprazine was administered in the dose range of 0.1–12 mg either in a fixed or flexible dose design. Most commonly, doses between 1.5 mg (in schizophrenia, bipolar depression, and major depression) and 6.0 mg (schizophrenia and mania) were used. Doses above 6.0 mg (9.0 and 12.0 mg) showed additional efficacy, but also increased side-effects, while doses below 1.5 mg showed no efficacy—therefore, the final approved dose range excludes these doses.

### 3.3. Patient Characteristics

The diagnosis was established through the different editions of the DSM and was confirmed using validated assessment tools for the respective disorders. Inclusion criteria involved cut-off values on these scales to recruit patients with a certain severity of their illness. The main exclusion criteria included other mental health disorders, acute risk for suicide, or any other relevant disorders that could have interfered with the results of the study. Details about inclusion and exclusion criteria were outlined in the respective publications (Table 3). During the studies, patients were allowed to use their regular non-centrally active medications and centrally active rescue medications that included benzodiazepines, anti-extrapyramidal symptom medications, and sleeping medications.

Patient numbers ranged between 118 per arm in a mania study [41] and 273 in the major depressive disorder study [52]. In most studies, patients were treated either with cariprazine or with a placebo. In two schizophrenia studies, an active comparator (risperidone 4 mg [38] and aripiprazole 10 mg [36]) was also used to assess sensitivity. In the major depressive studies, antidepressants had been used as base treatment before cariprazine or placebo add-on treatment was initiated [51,52]. In the schizophrenia primary negative symptom study, cariprazine was compared to risperidone—this was an active-controlled, superiority study that did not have a placebo arm [40]. Treatment periods ranged from 3 weeks in the mania studies to up to 92 weeks in the schizophrenia relapse prevention study.

### 3.4. Efficacy on Transdiagnostic Symptoms

#### 3.4.1. Positive Symptoms

In the RCTs with cariprazine, positive symptoms were measured in the schizophrenia and mania studies using the PANSS Total Score, PANSS-FSPS, and the YMRS Item 8 (‘Content’) score as assessment tools. In the depression studies (both bipolar I and major depression add-on), psychotic patients were excluded, and psychotic symptoms were not assessed during the study.

Cariprazine reduced positive symptoms in both schizophrenia and mania patients. Of the overall four studies in patients with acute schizophrenia, only one Phase 2 study had a negative outcome; however, it helped the design and conduct of subsequent Phase 2b/3 clinical trials. The other three showed positive results for all examined doses. In these latter pooled schizophrenia studies, at Week 6, statistically significant differences of cariprazine (1.5–9.0 mg/d) versus placebo were seen on the PANSS-FSPS (effect size (ES) = 0.37, *p* < 0.0001) [54]. Additionally, statistically significant differences of cariprazine versus placebo were seen in the two pooled, fixed-dose studies (MD-16 and MD-14) [36,38] for 3.0 mg/d (least square mean difference (LSMD) = −1.4, 95% CI [−2.2, −0.6], *p* = 0.0011, ES = 0.32); 4.5 mg/d (LSMD = −2.1, 95% CI [−3.2, −1.1], *p* = 0.0001, ES = 0.52) and the 6.0 mg/d (LSMD = −2.2, 95% CI [−3.3, −1.1], *p* < 0.0001, ES = 0.42) [54]. Numerical differences were also seen for 1.5 mg/d, although it did not reach statistical significance (LSMD = −0.7, 95% CI [−1.8, 0.4], *p* = 0.2365, ES = 0.25) [54].

In the pooled bipolar I mania studies, at Week 3, both low-dose (3.0–6.0 mg/d) and high-dose (6.0–12.0 mg/d) cariprazine significantly improved PANSS Total Scores compared to placebo (*p* < 0.001) [43]. Additionally, based on Item 8 (‘Content’) of the YMRS in the pooled mania studies, the difference in mean change from baseline to end (Week 3) was statistically significant in favor of cariprazine over placebo (LSMD: −0.8, 95% CI [−1.0 −0.5], *p* < 0.001) [59].

#### 3.4.2. Negative Symptoms

In the RCTs, negative symptoms were measured in the schizophrenia studies only, using the PANSS-FSNS and NSA-16. Cariprazine showed statistically significant effects in improving negative symptoms of schizophrenia.

Measuring negative symptoms in the general/acute schizophrenia population (potentially with high secondary negative symptoms), at Week 6, statistically significant differences versus placebo were seen for cariprazine on the PANSS-FSNS (with ES for the different doses ranging between 0.34 and 0.62, *p* < 0.0001) [54]. Additionally, in all studies, significant results were reported on the NSA-16 Total Score, except for MD-05 [37], where only one dose group (6–9 mg/d) was significant while the other (3–6 mg/d) was not.

When looking at a subpopulation of patients from the same acute population who experience predominantly negative symptoms, significant differences were found for cariprazine at doses of 1.5–3.0 mg/d (LSMD = −2.0, 95% CI [−3.6, −0.3], *p* = 0.0179, ES = 0.41), cariprazine 4.5–6.0 mg/d (LSMD = −3.4, 95% CI [−5.2, −1.7], *p* = 0.0002, ES = 0.71) as well as for risperidone (LSMD = −2.8, 95% CI [−5.0, −0.5], *p* = 0.0149, ES = 0.57) over placebo in the treatment of these symptoms [56]. However, no significant difference was observed for aripiprazole compared to placebo (LSMD = −1.0, 95% CI [−3.0, 1.0], *p* = 0.3265). At Week 6, the group receiving cariprazine at 4.5 mg/d showed a significantly greater reduction in PANSS-FSNS from baseline compared to the aripiprazole group (LSMD = −2.4, 95% CI [−4.5, −0.4], *p* = 0.0197, ES = 0.50). No significant difference was found between cariprazine at 4.5–6.0 mg/d and risperidone (LSMD = −0.7, 95% CI [−2.9, 1.6], *p* = 0.5464). After adjusting for changes in positive symptoms, cariprazine continued to show statistically significant differences versus placebo (1.5–3.0 mg/d: LSMD = −1.4, 95% CI [−2.7, −0.1], *p* = 0.0322; 4.5–6.0 mg/d: LSMD = −2.1, 95% CI [−3.5, −0.7], *p* = 0.0038), while risperidone (LSMD = −1.1, 95% CI [−2.8, 0.7], *p* = 0.2204) and aripiprazole (LSMD = −0.2 95% CI [−1.8, 1.3], *p* = 0.7635) did not [56].

Finally, in a specially designed study on primary negative symptoms of schizophrenia, cariprazine led to greater least squares mean changes in PANSS-FSNS from baseline to Week 26 than risperidone (LSMD = −1.46, 95% CI [−2.39, −0.53], *p* = 0.0022, ES = 0.31) [40].

#### 3.4.3. Cognitive Symptoms

Cariprazine improved cognitive symptoms in schizophrenia, mania, and depressed patients. In the schizophrenia studies, at Week 6, statistically significant differences of cariprazine (1.5–9.0 mg/d) versus placebo were seen on the PANSS Disorganized Thought Factor (*p* < 0.0001, ES = 0.47) [54]. Additionally, statistically significant differences of cariprazine versus placebo were seen in the two fixed-dose studies (MD-04 and MD-16) [36,38] for all doses (1.5 mg/d: LSMD = −1.2, 95% CI [−2.0, −0.5], *p* = 0.0009, ES 0.40; 3.0 mg/d: LSMD = −1.2, 95% CI [−1.7, −0.6], *p* < 0.0001, ES = 0.38; 4.5 mg/d: LSMD = −1.8, 95% CI [−2.5, −1.0], *p* < 0.0001, ES = 0.60; and 6.0 mg/d: LSMD = −1.7, 95% CI [−2.4, −1.0], *p* < 0.0001, ES = 0.49] [54].

In the pooled mania studies, at Week 3, statistically significant differences versus placebo were seen on Item 7 (language-thought disorder: LSMD = −0.3, 95% CI [−0.5, −0.2], *p* < 0.001, ES = 0.36) [59].

In the pooled bipolar depression studies, at Week 6, statistically significant differences versus placebo were seen on Item 6 (concentration difficulties: LSMD = −0.3, 95% CI [−0.5, −0.1], *p* < 0.001) [60].

Additionally, post hoc analyses were performed on bipolar I depression, mania, and schizophrenia studies using the MADRS, FAST, PANSS, and the CDR Attention Battery to measure cognitive symptoms [64]. LSMDs in changes from baseline to end were reported for specific patient subsets with varying levels of baseline cognitive symptoms. In patients with bipolar depression exhibiting at least mild cognitive symptoms, LSMDs showed significant differences for cariprazine versus placebo on MADRS Item 6 (across three studies: 1.5 mg/d = −0.5, *p* < 0.001; 3.0 mg/d = −0.2, *p* < 0.05) and on the FAST cognitive subscale (one study: 1.5 mg/d = −1.4, *p* = 0.0039). For those with bipolar mania and mild cognitive symptoms, the LSMD in the PANSS Disorganized Thought Factor Score was also significant for cariprazine versus placebo (three studies: −2.1, *p* = 0.001). In patients with schizophrenia experiencing high cognitive impairment, cariprazine 3.0 mg/d demonstrated improvements in attention power compared to placebo (*p* = 0.0080), while no significant effect was noted for the 6.0 mg/d dose. Additionally, enhancements in continuity of attention were observed for both cariprazine 3.0 mg/d (*p* = 0.0012) and 6.0 mg/d (*p* = 0.0073) on the CDR Attention Battery [64]. Negative results were only seen on the CTT in two schizophrenia studies [36,37].

Single-item scores on the MADRS are not available in the aMDD publications; hence, cognition based on the MADRS Item 6 in these studies is not available.

#### 3.4.4. Manic Symptoms

In the RCTs, manic symptoms were measured in three bipolar mania studies and for safety reasons in the bipolar I depression studies based on the YMRS Total Score. In the latter, little change was seen on the YMRS, indicating that patients did not switch to mania during the study [62].

Cariprazine reduced manic symptoms in the bipolar mania studies: all examined doses of cariprazine in all three studies were statistically significant vs. placebo: the LSMD for overall cariprazine vs. placebo was −5.35 (95% CI [−6.69, −4.01], *p* < 0.0001, ES = 0.54) [65]. Moreover, significant improvement in mean change from baseline to Week 3 was seen on all 11 individual YMRS symptom items in favor of cariprazine vs. placebo [59].

#### 3.4.5. Depressive Symptoms

In the RCTs, efficacy on depressive symptoms was measured in the schizophrenia studies (PANSS Anxiety/Depression Factor Score and Depression item), in four bipolar depression studies, and five major depression add-on studies (MADRS Total Score/items). Additionally, to monitor switching to depression, depressive symptoms were also monitored for safety reasons in the mania studies (MADRS Total Score/items) [62]. Overall, cariprazine improved depressive symptoms across populations.

From the four bipolar depression studies, MD-52 [44] was a failed Phase 2 study, which helped the design and conduct of subsequent Phase 2b/3 clinical trials. Pooled analyses of the latter three studies showed statistically significant improvement in depressive symptoms with cariprazine versus placebo on MADRS Total Scores and all individual MADRS items [60]: 1.5 mg/d (MADRS Total Score LSMD = −2.8, 95% CI [−4.1, −1.6], *p* < 0.001); 3 mg/d (LSMD = −2.4, 95% CI [−3.7, −1.2], *p* < 0.001) and pooled 1.5–3.0 mg/d dose (LSMD = −2.6, 95% CI [−3.7, −1.5], *p* < 0.001) [60].

In the major depressive disorder add-on studies, pooled data on MADRS could not be retrieved. Of the overall five studies, two individual studies showed statistically significant results versus placebo. In study 3111-301-001 [52], adjunctive cariprazine 1.5 mg/d compared with placebo resulted in significantly greater mean reductions in MADRS Total Score from baseline to Week 6 (LSMD = −2.5, 95% CI [−4.2, −0.9], *p* = 0.0025). Cariprazine 3.0 mg/d versus placebo reached numerically greater reductions in MADRS Total Score; however, this difference did not reach statistical significance (LSMD = −1.5, 95% CI [−3.2, 0.1], *p* = 0.0691) [52]. In MD-75 [51], patients taking cariprazine at doses of 2.0–4.5 mg/d showed significantly greater mean reductions in the MADRS Total Score compared to placebo by Week 2 and at all subsequent visits. By Week 8, the LSMD for the cariprazine 2–4.5 mg/day group versus placebo was –2.2 (95% CI [–3.7, –0.6], *p* = 0.0057). In contrast, the LSMD for the cariprazine 1.0–2.0 mg/d group at Week 8 was –0.9 (95% CI [–2.4, 0.6], *p* = 0.2404) [43].

In the pooled schizophrenia studies, significant improvement was observed on the PANSS Anxiety/Depression Factor Score (*p* < 0.01, ES = 0.21) and on the G6 item of Depression (*p* < 0.5) [54].

**Figure 2:**
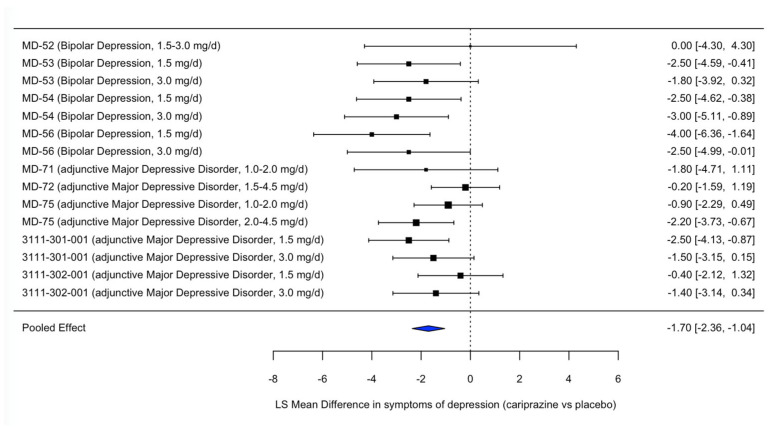
Forest plot of the efficacy of cariprazine versus placebo on depressive symptoms across psychiatric disorders. Footnote: The forest plot displays the least squares (LS) mean difference in MADRS Total Score between cariprazine and placebo, based on randomized controlled trials in bipolar depression and major depressive disorder. Negative values indicate greater improvement in depressive symptoms with cariprazine relative to placebo. The blue diamond represents the pooled effect estimate across studies, with its width corresponding to the 95% confidence interval. The vertical dashed line indicates the line of no effect (LSMD = 0).

A meta-analysis was conducted to interpret the overall efficacy of cariprazine on depressive symptoms as measured by the MADRS Total Score. The meta-analysis included 15 treatment arms from clinical trials of cariprazine in patients with bipolar depression and major depressive disorder. Several trials contributed multiple active dose groups compared to a shared placebo group, which was appropriately modelled using a multilevel structure. The pooled LS mean difference in depressive symptom change from baseline favored cariprazine over placebo (estimate = –1.70, SE = 0.34, *p* < 0.0001), with a 95% confidence interval of –2.36 to –1.04. Between-study heterogeneity was low and not statistically significant (Q = 16.68, *p* = 0.27; τ^2^ = 0.38). The results indicate that cariprazine has a robust and consistent effect in reducing depressive symptoms across patient populations with a depressive episode (Figure 2).

#### 3.4.6. Addiction Symptoms

Addiction symptoms were not assessed in the RCTs. In fact, known substance use disorder and/or positive urine drug screens at baseline were exclusionary and were not repeated during the studies, so potential occasional use of illicit drugs was not reassessed.

#### 3.4.7. Sleep Symptoms

In the RCTs, efficacy on sleep symptoms was not measured with dedicated scales. In the context of depression or mania, sleep was measured in the respective studies based on Item 4 of both the MADRS and the YMRS scales.

In the pooled bipolar mania studies, at Week 3, statistically significant differences versus placebo were seen on Item 4 (‘Sleep’) of the YMRS (LSMD = −0.3, 95% CI [−0.5, −0.2], *p* < 0.001) [59].

In the pooled bipolar depression studies, at Week 6, statistically significant differences versus placebo were seen on Item 4 (‘Reduced sleep’) on the MADRS in the 1.5–3.0 mg/d group (LSMD = −0.2, 95% CI [−0.4, −0.0], *p* = 0.04) [60].

Single-item scores on the MADRS are not available in the aMDD publications; hence, sleep based on the MADRS Item 4 in these studies is not available.

#### 3.4.8. Anxiety Symptoms

In the RCTs with cariprazine, anxiety symptoms were measured in the pooled schizophrenia studies using the PANSS Anxiety/Depression Factor Score/Item [54] and with MADRS Item 3 (‘Inner tension’) in the pooled bipolar depression studies. Additionally, and more specifically, anxiety was also assessed with the HAM-A scale in two bipolar depression studies [45,46] and three major depression studies [50,52,53]. In the aMDD studies, item analyses of MADRS scores were not retrievable.

In the framework of schizophrenia, statistically significant differences of cariprazine (1.5–9.0 mg/d) versus placebo were seen on the PANSS Anxiety/Depression Factor Score (*p* < 0.01, ES = 0.21) and on the G2 item of Anxiety (*p* < 0.1) [54].

In the pooled bipolar depression studies, at Week 6, statistically significant differences versus placebo were seen on Item 3 (‘Inner tension’) in the 1.5 mg/d dose group (LSMD = −0.2, 95% CI [−0.4, −0.0], *p* = 0.03) [60]. Additionally, cariprazine 1.5 and 3 mg/d were evaluated in patient subgroups with higher and lower baseline anxiety. Mean change from baseline versus placebo in HAM-A Total Score at Week 6 was statistically significant for cariprazine 1.5 mg/d in the higher anxiety subgroup (*p* = 0.0105) and cariprazine 3 mg/d in the lower anxiety subgroup (*p* = 0.0441) [61].

**Figure 3:**
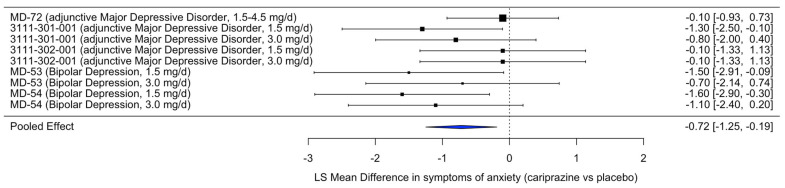
Forest plot of the efficacy of cariprazine versus placebo on anxiety symptoms across psychiatric disorders. Footnote: the forest plot displays the least squares (LS) mean difference in HAM-A Total Score between cariprazine and placebo, based on randomized controlled trials in bipolar depression and major depressive disorder. Negative values indicate greater improvement in anxiety symptoms with cariprazine relative to placebo. The blue diamond represents the pooled effect estimate across studies, with its width corresponding to the 95% confidence interval. The vertical dashed line indicates the line of no effect (LSMD = 0).

In the aMDD studies, HAM-A scores were assessed in three studies, but significant improvement was only recorded in one study for the 1.5 mg [52]. However, looking at the overall efficacy of cariprazine on anxiety symptoms as measured by the HAM-A at Week 6 or 8 in all bipolar depression and aMDD trials, a meta-analysis shows that the overall pooled effect is significant (Figure 3). The meta-analysis included nine treatment arms from trials assessing anxiety symptom change. Cariprazine again showed a statistically significant improvement over placebo (estimate = –0.72, SE = 0.27, *p* = 0.008), with a 95% confidence interval of –1.25 to –0.19. Heterogeneity was minimal and not statistically significant (*Q* = 8.23, *p* = 0.41; τ^2^ = 0.16). These results indicate that cariprazine has a moderate effect in alleviating anxiety symptoms across relevant patient populations.

#### 3.4.9. Hostility Symptoms

In the RCTs, hostility symptoms were measured in the schizophrenia and mania studies. Cariprazine reduced hostility symptoms in both indications [55,58].

In the pooled schizophrenia studies, at Week 6, statistically significant differences of cariprazine (1.5–9.0 mg/d) versus placebo were seen on the PANSS Uncontrolled Hostility/Excitement Factor Score (*p* < 0.0001, ES = 0.34). Additionally, statistically significant differences of cariprazine versus placebo were seen in the two fixed-dose studies (MD-04 and MD-16) [36,38] for all cariprazine doses (1.5 mg/d: LSMD = −0.9, 95% CI [−1.6, −0.2], *p* = 0.0076, ES = 0.39; 3.0 mg/d: LSMD = −0.7, 95% CI [−1.22, −0.2], *p* = 0.0057, ES 0.33; 4.5 mg/d: LSMD = −0.6, 95% CI [−1.2, 0.1], *p* = 0.0716, ES = 0.31 and the 6.0 mg/d: LSMD = −1.1, 95% CI [−1.8, −0.5], *p* = 0.0007, ES = 0.36) [54].

Furthermore, in a sub-analysis in patients exhibiting different levels of baseline hostility, the LSMD in the change from baseline to Week 6 on the P7 Hostility item was statistically significant for cariprazine over placebo (LSMD = –0.28, 95% CI [−0.41, –0.15], *p* < 0.0001) [55]. Notably, the degree of change for cariprazine was greater among participants with higher baseline hostility, with LSMD values compared to placebo for subgroups of P7 Hostility item ≥ 2, ≥3, and ≥4 being –0.32, –0.37, and –0.51, respectively (all *p* < 0.01) [55].

In the pooled bipolar mania studies, at Week 3, both YMRS Item 5 (‘Irritability’) and 9 (‘Disruptive-aggressive behaviors’) were statistically significant in favor of cariprazine over placebo (Irritability: LSMD: −0.8, 95% CI [−1.1, −0.6], *p* < 0.001; Disruptive behavior: LSMD = −0.7, 95% CI [−0.9, −0.5], *p* < 0.001) [59]. In fact, the largest effect sizes for cariprazine were noted on these two items (Irritability: 0.55; Disruptive–aggressive behavior: 0.49) [59]. In a subgroup analysis in patients with baseline score ≥ 2 on both the YMRS irritability and disruptive–aggressive behavior items, LSMD in change from baseline to Week 3 was statistically significant in favor of cariprazine versus placebo on both items (Irritability: LSMD = –0.93, *p* < 0.001; Disruptive–aggressive behavior: LSMD = −0.79, *p* < 0.001) [58]. In the same subgroup, patients were also examined on the change from baseline to treatment end in their PANSS P7 Hostility item scores. Statistically significant results were attained compared to placebo for both cariprazine dosage groups (3.0–6.0 mg/d: LSMD = –0.70; *p* < 0.0001; and 6.0–12.0 mg/d: LSMD = –0.49, *p* = 0.0002) [58].

#### 3.4.10. Self-Harm Symptoms

In the RCTs, individuals with suicidal tendencies were not included, which means that the impact of cariprazine on reducing suicidal symptoms could not be assessed. However, the C-SSRS was utilized in nearly all studies (except for MD-03 [35], MD-16 [38], MD-31 [41], and MD-71 [49]) to monitor suicidality across conditions such as schizophrenia, mania, bipolar depression, and MDD as a safety measure. This tracking ensured that any potential risks related to suicidality occurring during the study, either related to the disorder or due to side effects, were monitored. Additionally, based on the MADRS Item 10, suicidality was monitored in the bipolar depression studies.

Analyzing the data recorded on the C-SSRS in the single studies, no patient had suicidal behavior and most patients had no suicidal ideation either [36,37,39,40,42,43,45,46,47,51,52]. In the low number of patients who showed suicidal ideation, most wished to be dead but had no plan to act on it. The most severe ideation recorded was “Active suicidal ideation with some intent to act, without specific plans”.

In the pooled bipolar depression studies, at Week 6, statistically significant differences versus placebo were seen on Item 10 (Suicidal thoughts: in the 1.5–3.0 mg/d group: LSMD = −0.1, 95% CI [−0.1, −0.0], *p* = 0.04) [60].

Based on the above, cariprazine did not cause suicidality as a side-effect, and managed to keep patients stable. Despite their disorder, which often includes a risk of suicidality, the patients did not deteriorate.

## 4. Discussion

**Figure 4:**
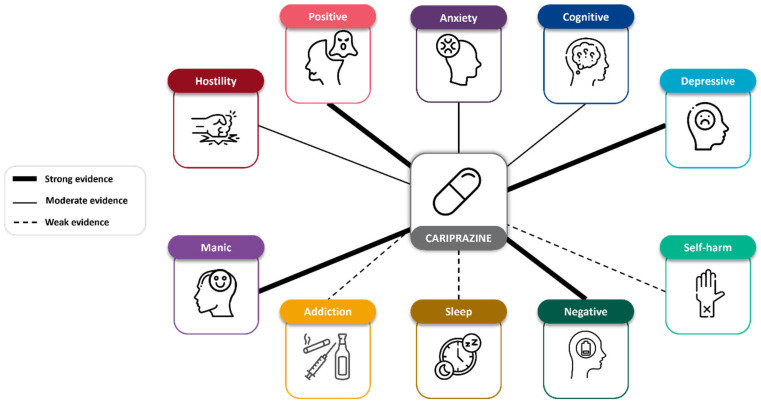
Schematic overview of symptom domains potentially impacted by cariprazine. Footnote: Strong evidence was defined as the presence of randomized controlled trials (RCTs) specifically designed to evaluate the symptom domain in question, conducted in a relevant and vulnerable patient population. Moderate evidence was assumed if findings were consistent across disorders but were derived from trials not specifically targeting the symptom domain or symptom-specific populations, or if symptom measurement relied on non-specific scales. Weak evidence was assumed when data came from non-RCTs or when limitations in design or measurement reduced interpretability.

The aim of this study is to assess the efficacy of cariprazine on ten transdiagnostic symptoms (positive, negative, cognitive, mania, depressive, addiction, anxiety, sleep, hostility, and self-harm) irrespective of underlying disorders. A comprehensive analysis of cariprazine’s efficacy revealed statistically significant improvements (compared to placebo or comparator antipsychotics) on seven of the ten transdiagnostic symptoms: positive, negative, cognitive, manic, depressive, anxiety, and hostility symptoms. Addiction symptoms were not assessed, as they were an exclusion criterion at baseline and were not monitored during the studies. Sleep symptoms were not assessed using specific scales, but based on single items derived from the YMRS and MADRS, cariprazine showed beneficial effects. Finally, although suicidality was assessed with a targeted rating scale (C-SRSS), patients included in the RCT trials were non-suicidal as per the inclusion/exclusion criteria. In this population, cariprazine did not cause suicidality as a side-effect and kept patients stable. Despite their disorder, which often includes a risk of suicidality, the patients did not deteriorate. An infographic summary of findings is presented below in Figure 4.

### 4.1. Positive, Manic, and Hostility Symptoms: The Role of Dopamine D_2_ Receptors

Positive, manic, and hostility symptoms are commonly linked to hyperdopaminergic states in the mesolimbic pathway. While various neurotransmitter systems—including GABA and glutamate—also contribute to these symptoms, most current antipsychotics act primarily on the D_2_ receptors [66]. Newer agents such as cariprazine, aripiprazole, and brexpiprazole are partial D_2_ agonists rather than antagonists [67]. Current guidelines do not differentiate between antipsychotics for addressing positive symptoms and a large meta-analysis comparing the efficacy of oral antipsychotics found that there are differences in the effectiveness of antipsychotics, but these differences tend to be gradual rather than discrete [68]. Instead, treatment choices should consider other aspects such as safety, adherence, long-term functioning, as well as formulation, dosing, onset of effect, and half-life.

Findings of the present systematic review endorse the utilization of cariprazine in positive, manic, and hostility symptoms [54,55,58,59]. Furthermore, there is considerable real-world evidence (RWE) to corroborate the results [69,70]. For instance, a study evaluating the long-term effectiveness and safety of cariprazine conducted in Italy found significant reductions in both positive and negative symptoms over 12 months, as measured by the PANSS [69]. Another pilot study aiming to evaluate the effectiveness and safety of adding cariprazine to clozapine treatment in patients with sub-optimal response found significant reductions in total, positive, and negative PANSS scores over three months. Specifically, the median total PANSS score decreased from 59 to 22.5, the positive PANSS score from 11.5 to 5.5, and the negative PANSS score from 15.5 to 3 [70].

### 4.2. Negative, Cognitive, and Addiction Symptoms: The Role of Dopamine D3 Receptors

Negative, cognitive, and addiction symptoms are associated with a dysregulation in the prefrontal cortex, a region crucial for planning, decision-making, and social behavior [71]. Additionally, dysregulation in the limbic system, which includes structures such as the hippocampus and amygdala, also leads to disturbances in emotional processing and memory, along with motivation and reward [71]. These crucial aspects of human behavior are mediated by the dopamine D_3_ receptors [72]. Importantly, cariprazine has the highest affinity to the D_3_ receptors among all available antipsychotics [72].

According to the findings of the present systematic review, cariprazine has the ability to improve negative and cognitive symptoms [40,54,56,59,60,64]. The efficacy of cariprazine on negative symptoms has been proven in a specifically designed study in patients with persistent, predominant negative symptoms [40], as well as in a subset of patients with predominant negative symptoms in the short-term efficacy studies [56]. Additionally, a meta-analysis, published in The Lancet, evaluated the effectiveness of various antipsychotic medications in treating negative symptoms of schizophrenia [73]. The study analysed 21 randomized controlled trials with 3451 participants and found that amisulpride was more effective compared to placebo and cariprazine compared to risperidone for the treatment of primary negative symptoms [73]. These were the only two drugs showing effects on predominant negative symptoms. The efficacy of cariprazine on cognitive symptoms, as assessed in the clinical studies, also shows promising results [54,59,60,64]. However, it must be noted that no RCTs have specifically assessed cariprazine for enhancing cognition, meaning that specific studies in patients with cognitive impairment have not been designed, and specific regulatory-requested outcome parameters, such as the Measurement and Treatment Research to Improve Cognition in Schizophrenia (MATRICS), have not been applied. Hence, further studies are needed to verify these positive preliminary results. Finally, the exclusion of patients with substance use disorders from the randomized clinical trials limits the ability to assess cariprazine’s transdiagnostic potential in this domain. To robustly validate the transdiagnostic properties of cariprazine on substance use and addiction, future research should include large-scale, prospective studies.

Looking at RWE, cariprazine’s efficacy is also supported by numerous studies [74,75,76]. A Latvian study investigated the effectiveness and safety of cariprazine in schizophrenia patients with negative symptoms who had not responded well to previous antipsychotic treatments [74]. Conducted over 16 weeks with 116 patients, the study found significant improvements in negative symptoms and overall clinical condition [74]. Specifically, there was a notable reduction in negative symptom scores, and over 70% of patients showed minimal to much improvement on the Clinical Global Impression—Improvement scale [74]. A Slovakian study further confirmed these findings: this was a 1-year-long longitudinal, prospective, multicentric cohort study that aimed to observe the treatment and psychosocial functioning of schizophrenia patients with predominant negative symptoms [75]. The study demonstrated significant improvements in negative symptoms and overall functioning with cariprazine, both as monotherapy and in combination with other treatments [75]. Notably, most patients were on polytherapy, with cariprazine as the common component. The study concluded that with appropriate treatment strategies, improvements in negative symptoms and daily functioning are achievable in schizophrenia outpatients [75]. Additionally, a pilot study with a 6-month follow-up aimed to evaluate the efficacy of cariprazine in treating negative symptoms in patients with early psychosis [76]. Conducted over six months, the case series involved ten patients with prominent negative symptoms [76]. The results revealed a substantial reduction in negative symptoms, as the mean PANSS negative score dropped from 26.3 to 10.6 [76]. Albeit RCTs are missing, a cohort study and various case reports are available to support the effectiveness of cariprazine on addiction symptoms [77,78,79,80,81,82,83,84,85,86,87,88]. In a study by Szerman et al. [77], the authors examined the use of cariprazine for treating dual disorders, specifically comorbid substance use disorder and schizophrenia. Cariprazine treatment led to significant improvements in schizophrenia symptoms, with a change of −47.88 points on the PANSS (*p* < 0.0001). Additionally, cannabis use and dependence decreased, as evidenced by a −7.0 point change on the Cannabis Abuse Screening Test (*p* < 0.0001) and a −7.88 point change on the Severity of Dependence Scale (*p* < 0.0001). These findings suggest that cariprazine is effective for patients with schizophrenia and cannabis use disorder. This is further supported by case reports describing cariprazine’s efficacy in reducing the craving and substance use in patients consuming methamphetamine, cocaine, cannabis, alcohol, and tobacco [78,79,80,81,82,83,84,85,86,87,88]. Consequently, current guidelines suggest cariprazine and other partial agonists as first-line treatment in maintenance settings and as second-line in acute settings in patients with substance use disorder comorbidity [89].

### 4.3. Depressive, Anxiety, and Self-Harm Symptoms: The Role of Serotonin 5-HT_1A_ and 5-HT_2A_ Receptors

The molecular basis of depression, anxiety, and suicidality involves complex interactions among various neurotransmitters and receptors. There are some overlapping mechanisms that are involved in all three conditions. Serotonin receptors, particularly 5-HT_1A_ and 5-HT_2A_, play an important role [24], along with dopamine D_2_ and D_3_ receptors, which are implicated in the reward system and motivation—these neurotransmitter systems are often disrupted in depression [90] and may also play a role in suicidality [91]. GABA-A receptors are critical for inhibitory neurotransmission, and their dysfunction can lead to increased anxiety [71]. In suicidality, in addition to serotonin and dopamine pathways, the hypothalamic–pituitary–adrenal (HPA) axis is involved [91]. Abnormalities in this stress response system are often found in individuals with suicidal behavior [91]. Cariprazine’s antidepressant and anxiolytic effects are primarily attributed to its partial agonist activity at dopamine D_3_, D_2_, and serotonin 5-HT_1A_ receptors, as well as its antagonist activity at serotonin 5-HT_2B_ and 5-HT_2A_ receptors [92].

The findings of the systematic review and meta-analysis indicated the capacity of cariprazine to improve depressive and anxiety symptoms. Indeed, in a recently published post hoc analysis [93], the effects of adjunctive cariprazine on anxiety symptoms in adults with MDD and varying levels of baseline anxiety were studied. Among 751 patients, cariprazine 1.5 mg/d significantly reduced anxiety symptoms at Week 6, especially in those with elevated baseline anxiety, as measured by the HAM-A scores. The results suggest that adjunctive cariprazine may be effective in alleviating anxiety symptoms in MDD patients, regardless of their initial anxiety severity. The anti-suicidal effects of cariprazine, however, are not well-understood. Results of the above clinical studies suggest that cariprazine does not induce suicidality, but systematic examinations in the vulnerable suicidal populations are lacking.

A recent RWE study [94] in adults with moderate-to-severe major depressive disorder or bipolar I depression evaluated the effectiveness of cariprazine as adjunctive and monotherapy on depressive symptoms in a real-world setting as measured by the Patient Health Questionnaire-9 (PHQ-9). Patients showed clinically meaningful reductions in PHQ-9 scores over 2, 6, and 12 months. Improvements were observed in both aMDD (mean reductions up to 4.1 points) and BP-I (up to 6.9 points), with greater effects seen in sensitivity analyses. While these findings support clinical trial data, they also highlight cariprazine’s sustained effectiveness on depressive symptoms in real-world practice. Furthermore, there is a case report of a suicidal adolescent patient who benefited from cariprazine, leading authors to suggest its potential usefulness in such cases [95].

### 4.4. Sleep Symptoms

Partial agonist activity at the dopamine D_2_-D_3_ receptors combined with individual patient variability can result in mixed effects on sleep, causing potentially sedation or insomnia [96]. Indeed, in some patients, cariprazine can lead to increased dopaminergic activity, which may result in heightened alertness and difficulty sleeping. Conversely, in other patients, the partial agonist activity can lead to a net inhibitory effect on dopaminergic pathways, particularly if their baseline dopaminergic activity is high, which can result in sedation. Genetic differences in dopamine receptor expression and function can also influence how a patient responds to cariprazine, contributing to either insomnia or sedation. Besides the effects on dopamine, cariprazine has no meaningful affinity to any receptors that regulate sleep [71,97,98,99]. Based on the results of the systematic review, however, cariprazine has shown benefits on sleep in mania and depression studies (where sleep is generally disturbed based on the disorder). In general, cariprazine is considered to be a rather activating, rather than a sedative drug, which might also underline its good efficacy in addressing amotivation and improving overall functioning [100].

It is important to note, however, that both insomnia and sedation can impact quality of life [101]. Some may find insomnia is more disruptive due to its impact on sleep quality, while others may be more troubled by the effects of sedation on alertness and daily functioning [101]. Importantly, persistent sedation has been associated with poor treatment adherence [101]. Insomnia occurs more frequently than sedation as a common side effect of cariprazine [100]. In the presented studies, sleep was not assessed with specific scales or methods; hence, the results must be interpreted with caution: sleep symptoms, as assessed by the YMRS and MADRS, have improved, while side effects of insomnia have been reported. Furthermore, cariprazine caused little sedation, which might have potential benefits concerning daily functioning, cognition, and overall treatment adherence.

### 4.5. Safety Considerations

Although the aim of the study was to assess the efficacy of cariprazine on ten transdiagnostic symptoms, highlighting the importance of safety is also crucial. Based on available literature, the safety profile of cariprazine within the approved dose range does not differ dramatically between bipolar disorder, major depressive disorder and schizophrenia, though certain nuances are worth noting: cariprazine is generally well-tolerated in all populations when used within the approved dose ranges (1.5–3 mg/d for bipolar depression and aMDD, 1.5–6 mg/d for schizophrenia, and 3–6 mg/day for manic/mixed episodes in bipolar I disorder). Generally, the most commonly reported side effects are EPS and akathisia, insomnia, and gastrointestinal symptoms. These appear in all populations, but slight variations in incidence rates depending on the disorder and dose apply [35,36,37,38,40,41,42,43,44,45,46,47,49,50,51,52,53,102]. Concerning gastrointestinal side effects, such as constipation, nausea, dyspepsia, and vomiting, the difference is more pronounced: these occur less frequently with schizophrenia and more with bipolar disorder and aMDD. Some of these commonly described side effects, such as akathisia and insomnia, might have impacts on the efficacy of cariprazine on transdiagnostic symptoms, specifically on self-harm, anxiety, and sleep.

Akathisia has been described in the literature as potentially worsening self-harm [103]. To address the potential for a relationship between suicidality and akathisia, in a previously conducted analysis [100], the incidence of C-SSRS suicidality or suicidality related treatment emergent adverse events (TEAEs) was evaluated in the subset of patients with a TEAE of akathisia, restlessness, or treatment-emergent akathisia as measured by the Barnes Akathisia Rating Scale (BARS) score ≤ 2 at baseline and >2 postbaseline in the schizophrenia studies. No relationship between suicidal tendency and akathisia/restlessness TEAEs or BARS-defined akathisia was observed. In the overall pooled schizophrenia studies, rates of any suicidality in patients with akathisia/restlessness were comparable between the placebo and cariprazine groups; rates of suicidal ideation in patients with akathisia/restlessness were slightly higher for both placebo- and cariprazine-treated patients, with most instances categorized in the least severe category (“wish to be dead”).

Furthermore, akathisia has also been linked to anxiety [66,71]. Akathisia and anxiety share overlapping clinical features, such as inner restlessness, agitation, and psychomotor symptoms, which can lead to diagnostic confusion [66,71]. Akathisia, a movement disorder often induced by antipsychotic medications, is characterized by a subjective sense of motor restlessness and a compelling urge to move [66,71]. Anxiety, on the other hand, is a psychiatric symptom complex that can also involve restlessness but is primarily driven by emotional distress [66,71]. The interaction between the two is bidirectional and potentially amplifying: anxiety can heighten the subjective distress associated with akathisia, while akathisia can provoke or worsen anxiety symptoms due to its intense, uncontrollable restlessness [66,71]. For cariprazine, akathisia has been described as a dose-dependent side effect, with higher doses potentially causing more akathisia. With higher doses, anxiety is also a common side effect observed in the schizophrenia trials [100]. Hence, while low doses of cariprazine, especially the 1.5 mg/d, seem to have beneficial effects in controlling anxiety [61,93], higher doses are causing anxiety, potentially through the increase of akathisia [100].

### 4.6. Limitations and Future Research

The present study has several limitations. First, systematic reviews are inherently dependent on the quality and availability of the included studies. If the primary studies are biased or of low quality, the reliability of the review’s conclusions can be compromised. However, in this analysis, all included studies were randomized controlled trials—the gold standard in clinical research—suggesting that overall study quality is not a concern. Second, publication bias is a common issue, as studies with negative or null results are less likely to be published. This concern is partially mitigated here, as all known development studies on cariprazine have been published. Third, heterogeneity in study populations, interventions, or outcome measures can complicate interpretation. However, since the goal of this review was to evaluate the transdiagnostic potential of cariprazine, such heterogeneity was expected and even desirable, and is therefore not considered to be a limitation in this context. That said, the assessment of symptoms could be improved. Ideally, symptoms would have been measured using a transdiagnostic scale—such as the TGI-P—and tracked prospectively. Instead, the studies relied on traditional, disorder-specific rating scales, which were then reinterpreted for the purposes of this review. Fourth, regarding the limitations of the meta-analysis, although all included analyses used consistent outcome measures (MADRS for depression and HAM-A for anxiety), variations in study design, treatment duration, and concurrent medications may have introduced unaccounted heterogeneity. Furthermore, while the results were derived from randomized controlled trials, the analyses were post hoc and may not have been specifically powered to evaluate changes in depressive or anxiety symptoms as primary outcomes.

To robustly validate the transdiagnostic properties of cariprazine, future research should include large-scale, prospective studies employing transdiagnostic symptom measures. Particular attention should be given to patient populations who are underrepresented in existing studies, including those with suicidality, anxiety disorders, substance use disorders, cognitive impairment, and various sleep disorders. This could pave the road towards a better understanding of the implications of the transdiagnostic concept and more personalized patient care.

## 5. Conclusions

Results indicate that cariprazine shows potential in addressing a broad range of symptom domains across psychiatric conditions.

## Figures and Tables

**Table 1:** Assessment of positive, negative, cognitive, mania, depressive, addiction, anxiety, sleep, hostility, and self-harm symptoms based on clinical assessment tools commonly used in the included trials.

	disorders	Bipolar Depression	Bipolar Mania	Major Depression	Schizophrenia
symptoms					
**Positive**		-	YMRS Item 8: content PANSS Total	-	PANSS FSPS
**Negative**		-	-	-	PANSS-FSNS NSA-16
**Cognitive**		MADRS Item 6: concentration difficulties FAST cognition	YMRS Item 7: language-thought disorder PANSS-disorganized factor score	-	PANSS-disorganized factor score CDR CTT
**Depressive**		MADRS Total Score	-	MADRS Total Score	PANSS anxiety/depression Factor score Depression (G6)
**Mania**		-	YMRS Total Score	-	-
**Addiction**		-	-	-	-
**Anxiety**		MADRS Item 3: inner tension HAMA	-	HAMA	PANSS anxiety/depression Factor score Anxiety (G2)
**Sleep**		MADRS Item 4: reduced sleep	YMRS Item 4: sleep	-	-
**Hostility**		-	YMRS Item 5: irritability, item YMRS Item 9: disruptive-aggressive behavior	-	PANSS hostility factor score Hostility item (P7)
**Self-harm**		MADRS Item 10: suicidal thoughts C-SSRS	C-SSRS	C-SSRS	C-SSRS

YMRS, Young-Mania Rating Scale; PANSS, Positive and Negative Syndrome Scale; PANSS-FSPS, Positive and Negative Syndrome Scale—Factor Score for Positive Symptoms; PANSS-FSNS, Positive and Negative Syndrome Scale—Factor Score for Negative Symptoms; NSA-16, Negative Symptom Assessment-16; MADRS: Montgomery–Åsberg Depression Rating Scale; FAST, Functioning Assessment Short Test; CDR, Cognitive Drug Research Battery; CTT, Color Trail Test; HAMA, Hamilton Anxiety Rating Scale; C-SSRS, Columbia-Suicide Severity Rating Scale.

**Table 2:** PANSS Marder factor scores.

Factor Score for Negative Symptoms (FSNS)		Factor Score for Positive Symptoms (FSPS)		Factor Score for Disorganized Thought		Factor Score for Uncontrolled Hostility/Excitement		Factor Score for Anxiety/Depression	
N1	Blunted affect	P1	Delusions	N5	Difficulty in abstract thinking	G14	Poor impulse control	G2	Anxiety
N2	Emotional withdrawal	P3	Hallucinatory behavior	G5	Mannerisms and posturing	P4	Excitement	G3	Guilt feelings
N3	Poor rapport	P5	Grandiosity	G10	Disorientation	P7	Hostility	G4	Tension
N4	Passive social withdrawal	P6	Suspiciousness	G11	Poor attention	G8	Uncooperative-ness	G6	Depression
N6	Lack of spontaneity	N7	Stereotyped thinking	G13	Disturbance of volition				
G7	Motor retardation	G1	Somatic concern	G15	Preoccupation				
G16	Active social avoidance	G9	Unusual thought content	P2	Conceptual disorientation				
		G12	Lack of judgement						

**Table 3:** List of included randomized clinical trials.

	Author, Year, Internal Code Reference	Design	Title	Indication	Transdiagnostic Symptom *
1	Durgam, 2016 (MD-03) [35]	Multicenter, randomized, double-blind, placebo-controlled, 6-week study	Cariprazine in the treatment of schizophrenia: A proof-of-concept trial	Schizophrenia	Positive Negative
2	Durgam, 2015 (MD-04) [36]	Multicenter, randomized, double-blind, placebo-controlled, 6-week study	Cariprazine in acute exacerbation of schizophrenia: A fixed-dose, phase 3, randomized, double-blind, placebo- and active-controlled trial	Schizophrenia	Positive Negative Cognitive Self-harm Depressive *Anxiety* *Hostility*
3	Kane, 2015 (MD-05) [37]	Multicenter, randomized, double-blind, placebo-controlled, 6-week study	Efficacy and safety of cariprazine in acute exacerbation of schizophrenia: Results from an international, phase III clinical trial	Schizophrenia	Positive Negative Cognitive Self-harm *Depressive* *Anxiety* *Hostility*
4	Durgam, 2014 (MD-16) [38]	Multicenter, randomized, double-blind, placebo-controlled, 6-week study	An evaluation of the safety and efficacy of cariprazine in patients with acute exacerbation of schizophrenia: A phase II, randomized clinical trial	Schizophrenia	Positive Negative *Cognitive* *Depressive* *Anxiety* *Hostility*
5	Durgam, 2016 (MD-06) [39]	Multicenter, randomized, double-blind, placebo-controlled, up to 92-week study	Long-term cariprazine treatment for the prevention of relapse in patients with schizophrenia: A randomized, double-blind, placebo-controlled trial	Schizophrenia	Positive Negative Self-harm
6	Németh, 2017 (005) [40]	Multicenter, randomized, double-blind, active-controlled, 26-week study in negative symptoms	Cariprazine as monotherapy for the treatment of predominant negative symptoms in patients with schizophrenia: A randomized, double-blind, active-comparator controlled trial	Schizophrenia	Negative Cognitive Self-harm
7	Durgam, 2015 (MD-31) [41]	Multicenter, randomized, double-blind, placebo-controlled, 3-week study	The efficacy and tolerability of cariprazine in acute mania associated with bipolar I disorder: a phase II trial	Bipolar Mania	Manic Positive Cognitive Hostility Sleep
8	Sachs, 2015 (MD-32) [42]	Multicenter, randomized, double-blind, placebo-controlled, 3-week study	Cariprazine in the treatment of acute mania in bipolar I disorder: A double-blind, placebo-controlled, phase III trial	Bipolar Mania	Manic Self-harm *Positive* *Cognitive* *Hostility* *Sleep*
9	Calabrese, 2015 (MD-33) [43]	Multicenter, randomized, double-blind, placebo-controlled, 3-week study	Efficacy and safety of low- and high-dose cariprazine in patients with acute and mixed mania associated with bipolar I disorder	Bipolar Mania	Manic Positive Cognitive Hostility Sleep Self-harm
10	Yatham, 2020 (MD-52) [44]	Multicenter, randomized, double-blind, placebo-controlled, 8-week study	Evaluation of cariprazine in the treatment of bipolar I and II depression: A randomized, double-blind, placebo-controlled, phase 2 trial	Bipolar Depression	Depressive Self-harm
11	Earley, 2020 (MD-53) [45]	Multicenter, randomized, double-blind, placebo-controlled, 6-week study	Efficacy and safety of cariprazine in bipolar I depression: A double-blind, placebo-controlled phase 3 study	Bipolar Depression	Depressive Self-harm *Anxiety* *Sleep* *Cognitive*
12	Earley, 2019 (MD-54) [46]	Multicenter, randomized, double-blind, placebo-controlled, 6-week study	Cariprazine treatment of bipolar depression: A randomized, double blind, placebo-controlled phase 3 study	Bipolar Depression	Depressive Self-harm *Anxiety* *Sleep* *Cognitive*
13	Durgam, 2016 (MD-56) [47]	Multicenter, randomized, double-blind, placebo-controlled, 8-week study	An 8-week randomized, double-blind, placebo-controlled evaluation of the safety and efficacy of cariprazine in patients with bipolar I depression	Bipolar Depression	Cognitive Depressive Anxiety Sleep Self-harm
14	McIntyre, 2024 (MD-25) [48]	Multicenter, randomized, double-blind, placebo-controlled up to 39 weeks study	Cariprazine as a maintenance therapy in the prevention of mood episodes in adults with bipolar I disorder	Bipolar Disorder both episodes	Depressive Manic Self-harm
15	Fava, 2018 MD-71 [49]	Multicenter, randomized, double-blind, placebo-controlled, 8-week study	Efficacy of adjunctive low-dose cariprazine in major depressive disorder: a randomized, double-blind, placebo-controlled trial	Major Depression	Depressive Self-harm
16	Earley, 2018 MD-72 [50]	Multicenter, randomized, double-blind, placebo-controlled, 8-week study	Cariprazine augmentation to antidepressant therapy in major depressive disorder: Results of a randomized, double-blind, placebo-controlled trial	Major Depression	Depressive Self-harm
17	Durgam, 2016 (MD-75) [51]	Multicenter, randomized, double-blind, placebo-controlled, 8-week study	Efficacy and safety of adjunctive cariprazine in inadequate responders to antidepressants: A randomized, double-blind, placebo-controlled study in adult MDD patients	Major Depression	Depressive Self-harm
18	Sachs, 2023 (3111-301-001) [52]	Multicenter, randomized, double-blind, placebo-controlled, 6-week study	Adjunctive cariprazine for the treatment of patients with major depressive disorder: A randomized, double-blind, placebo-controlled phase 3 study	Major Depression	Depressive Anxiety Self-harm
19	Riesenberg, 2023 (3111-302-001) [53]	Multicenter, randomized, double-blind, placebo-controlled, 6-week study	Cariprazine for the adjunctive treatment of major depressive disorder in patients with inadequate response to antidepressant therapy: Results of a randomized, double-blind, placebo-controlled study	Major Depression	Depressive Anxiety Self-harm
20	Marder, 2019 [54]	Pooled post hoc of 3 RCT	Efficacy of cariprazine across symptom domains in patients with acute exacerbation of schizophrenia: Pooled analyses from 3 phase II/III studies	Schizophrenia	Positive Negative Cognitive Depressive Anxiety Hostility
21	Citrome, 2016 [55]	Pooled post hoc of 3 RCT	The effect of cariprazine on hostility associated with schizophrenia: Post hoc analyses from 3 randomized controlled trials	Schizophrenia	Hostility
22	Earley, 2019 [56]	Pooled post hoc of 3 RCT	Efficacy of cariprazine on negative symptoms in patients with acute schizophrenia: A post hoc analysis of pooled data	Schizophrenia	Negative
23	Fleischhacker, 2019 [57]	Post hoc of the 005 study	The efficacy of cariprazine in negative symptoms of schizophrenia: Post hoc analyses of PANSS individual items and PANSS-derived factors	Schizophrenia	Negative Positive Depressive Cognitive Anxiety Hostility
24	Citrome, 2024 [58]	Post hoc of 3 RCT	Effects of cariprazine on reducing symptoms of irritability, hostility, and agitation in patients with manic or mixed episodes of bipolar I disorder	Bipolar mania	Hostility
25	Vieta, 2015 [59]	Post hoc of 3 RCT	Effect of cariprazine across the symptoms of mania in bipolar I disorder: Analyses of pooled data from phase II/III trials	Bipolar mania	Manic Positive Cognitive Sleep Hostility
26	Yatham, 2020 [60]	Pooled post hoc of 3 RCT	Broad efficacy of cariprazine on depressive symptoms in bipolar disorder and the clinical implications	Bipolar Depression	Depressive Cognitive Anxiety Sleep Self-harm
27	Jain, 2024 [61]	Pooled post hoc of 2 RCT	Efficacy of cariprazine in patients with bipolar depression and higher or lower levels of baseline anxiety: a pooled post hoc analysis	Bipolar Depression	Anxiety
28	Vieta, 2024 [62]	Pooled post hoc of 6 RCT	Full-spectrum efficacy of cariprazine across manic and depressive symptoms of bipolar I disorder in patients experiencing mood episodes: Post hoc analysis of pooled randomized controlled trial data	Bipolar Disorder both episodes	Depressive Manic
29	Citrome, 2024 [63]	Pooled post hoc of 5 RCT	Adjunctive cariprazine for the treatment of major depressive disorder: Number needed to treat, number needed to harm, and likelihood to be helped or harmed	Major Depression	Depressive
30	McIntyre, 2023 [64]	Pooled post hoc in all indications	The efficacy of cariprazine on cognition: a post hoc analysis from phase II/III clinical trials in bipolar mania, bipolar depression, and schizophrenia	Bipolar Disorder both episodes, Schizophrenia	Cognitive

Footnote: * Italicized elements indicate that the original study did not report these symptoms directly, but the study was included in pooled analyses where these symptom domains were subsequently analyzed and reported on.

## Data Availability

The data used in this study were derived from clinical trial datasets. Requests for clarification regarding the data may be directed to the corresponding author.

## Supplementary Materials

The following supporting information can be downloaded at https://www.mdpi.com/article/10.3390/ph18070995/s1, Table S1: Mapping process from symptom to instrument (Table 1).
