# Supplementary material for: Transdiagnostic Efficacy of Cariprazine: A Systematic Review and Meta-Analysis of Efficacy Across Ten Symptom Domains
Source: Pharmaceuticals (Basel). 2025 Jul 2;18(7):995. doi: 10.3390/ph18070995 (PMC12298362; doi:10.3390/ph18070995)
Supplement: Supplementary file 1 [file pharmaceuticals-18-00995-s001.zip › pharmaceuticals-3675496-supplementary.pdf]

Supplementary material

"Supplement 1 - Mapping process from symptom to instrument (Table 1)

The process of mapping specific symptom domains (e.g. cognitive, anxiety, depression, etc.) to assessment tools or their sub-items (e.g. YMRS Item 7, HAMA, MADRS total score, etc.) as presented in the manuscript in Table 1 was a key methodological step. The following methodology was applied:

1. Raw data extraction (i.e. data pulled from each study): For each study, outcome parameters were systematically identified, including all reported primary, secondary, and additional endpoints. A list of all published outcome parameters is provided in **List 1**.
2. These were then analyzed in relation to their relevance to transdiagnostic symptom domains with the following steps:
    a. Does the scale measure any symptoms?
    b. If yes, does it measure any of the 10 symptoms in question?
    c. If yes, scales were added to the table (**Table 1**)
3. Ambiguous or overlapping items were identified and handled as follows:
    a. validated instruments for certain symptom domains were preferred over single items
    b. when more than one scale measured the same symptom domain:
        i. primary endpoints were preferred over other endpoints as sample size calculations of the original studies were based on the primary endpoints
        ii. PANSS factors scores were preferred over PANSS subscale scores based on Marder SR, Davis JM, Chouinard G. The effects of risperidone on the five dimensions of schizophrenia derived by factor analysis: combined results of the North American trials. J Clin Psychiatry. 1997 Dec;58(12):538-46. doi: 10.4088/jcp.v58n1205. Erratum in: J Clin Psychiatry 1998 Apr;59(4):200. PMID: 9448657.
        iii. when an efficacy assessment tool was only used as a safety parameter in the studies and did not add any insights on the efficacy on a certain symptom domain (e.g. MADRS in the mania studies or CDSS in the negative symptom study), it was not considered.
        iv. however, when an efficacy assessment tool was used to monitor safety (e.g., C-SSRS) but addressed a symptom domain otherwise not measured (here: self-harm) it was considered.
4. **Table 1** in the manuscript is the final table as used for the analysis.

List 1: Raw data extraction
Schizophrenia

- MD-03: PANSS Total; CGI-S; CGI-I; Abnormal Involuntary Movement Scale (AIMS); Barnes Akathisia Scale (BARS) Items 1–3; Simpson–Angus Scale (SAS) // PANSS negative score; PANSS positive score
- MD-04: PANSS Total; CGI-S; 16-item Negative Symptom Assessment (NSA-16) Total Score; CGI-I; Schizophrenia Quality of Life Scale-Revision 4 (SQLSR4) Total Score; Cognitive Drug Research (CDR) Attention Battery; Color Trails Test (CTT); C-SSRS; AIMS; BARS; SAS // PANSS positive subscale; PANSS negative subscale; PANSS general psychopathology subscale; PANSS cognitive subscale; PANSS depression cluster; NSA-16 global negative symptom rating; SQLS-R4 vitality score; SQLS-R4 psychosocial score; CDR—power of attention score (msec); CDR—continuity of attention score; PANSS response
- MD-05: PANSS Total; CGI-S; CGI-I; NSA-16; SQLS-R4; CDR Attention Battery Tests; Color Trails Test; C-SSRS; AIMS; BARS items 1 to 3; SAS // PANSS positive; PANSS negative; NSA Global Negative Symptom Rating; SQLS-R4 psychosocial; SQLS-R4 vitality

- MD-16: PANSS Total; CGI-S; NSA-16; CGI-I; AIMS; BARS; SAS // PANSS positive subscale; PANSS negative subscale; NSA-16 Global Negative Symptom; PANSS responders
- MD-06: time to relapse; PANSS Total; CGI-S; ~~**PANSS items**~~; NSA-16 Total; PSP, SAS, BARS, C-SSRS // PANSS positive subscale; PANSS negative subscale
- MD-005: PANSS-FSNS; PSP Total; PANSS-FSPS; Calgary Depression Scale for Schizophrenia (CDSS) Total; SAS items 1-8 // PANSS Total; PANSS positive subscale; PANSS negative subscale; PANSS general psychopathology subscale; PSP self-care; PSP socially useful activities; PSP personal and social relationships; PSP disturbing and aggressive behaviour, C-SSRS

Bipolar Mania

- MD-31: YMRS; CGI-S; CGI-I; MADRS; PANSS; AIMS; BARS; SAS // YMRS single items
- MD-32: YMRS; CGI-S; CGI-I; MADRS; PANSS; C-SSRS; AIMS; BARS; SAS // YMRS response; YMRS remission
- MD-33: YMRS; CGI-S; CGI-I; MADRS; PANSS; BARS; SAS; AIMS; C-SSRS // MADRS response; YMRS remission, YMRS single items

Bipolar Depression

- MD-52: MADRS Total; CGI-I; CGI-S; HAMD-24; HAMD-17; MADRS response; MADRS remission; CGI-I response; HAMD-17 remission; BARS; AIMS; SAS; C-SSRS
- MD-53: MADRS Total; CGI-S; HAMD-17; HAM-A; Quick Inventory of Depressive Symptomatology – Self-Report (QIDS-SR16); BARS; AIMS; SAS; C-SSRS // MADRS response; MADRS remission; HAMD-17 remission
- MD-54: MADRS; CGI-S; HAM-D; HAM-A; QIDS-SR16; MADRS response; MADRS remission; HAM-D remission; BARS; AIMS; SAS; C-SSRS
- MD-56: MADRS; CGI-S; HAM-D; C-SSRS; BARS; AIMS; SAS; FAST // MADRS response; MADRS remission; HAM-D remission; MADRS single items

Major Depression

- MD-71: MADRS; CGI-I; CGI-S; HAMD-17 Total; HAMD-24 Total; HAMD-24 subsections; HAMD-17 subsections; MADRS response; MADRS remission; HAMD-17 remission; CGI-I response; AIMS; BARS; SAS
- MD-72: MADRS; SDS; CGI-S; CGI-I; HAMD-17; HAM-A; SDS subscales (work, social, family life); CGI-I response; MADRS response; MADRS remission; C-SSRS; AIMS; BARS; SAS
- MD-75: MADRS; SDS; CGI-S; SDS subscales (work, social, family); MADRS response; CGI-I response; MADRS remission; BARS; AIMS; SAS; C-SSRS // CGI-I
- 301: MADRS; CGI-S; MADRS response; MADRS remission; HAMD-17; HAM-A; CGI-I; CGI-I response; C-SSRS; BARS; AIMS; SAS // HAM-A response; HAM-A remission
- 302: MADRS; CGI-S; CGI-I; HAMD-17; HAM-A; AIMS; BARS; SAS; C-SSRS // MADRS response; MADRS remission; CGI-I response
